# First molecular screening of *Plasmodium* species in ungulates from Southern Brazil

**DOI:** 10.1186/s13104-018-3638-5

**Published:** 2018-07-31

**Authors:** Leonilda Correia dos Santos, Lilian de Oliveira Guimarães, Ana Laura Grazziotin, Wanderlei de Morais, Zalmir Silvino Cubas, Marcos José de Oliveira, Rafael Felipe da Costa Vieira, Alexander Welker Biondo, Karin Kirchgatter

**Affiliations:** 1 Department of Veterinary Medicine, Universidade Federal do Paraná, Curitiba, PR Brazil; 2 Engineering and Exact Sciences Center, Universidade Estadual do Oeste do Paraná, Foz Do Iguaçu, PR 85870-650 Brazil; 3 Malaria Research Center, Superintendence for Endemic Disease Control, Institute of Tropical Medicine, University of São Paulo, São Paulo, SP Brazil; 4 Department of Biomedical Engineering, College of Engineering, University of Iowa, Iowa City, IA USA; 5 Bela Vista Biological Sanctuary, Itaipu Binational Hydroelectric Power Plant, Foz Do Iguaçu, PR Brazil

**Keywords:** Malaria, PCR, Deer, Zoo animals, Brazil

## Abstract

**Objective:**

Despite malaria epidemiology has been extensively studied in primates, few studies were conducted in ungulates. After half a century without descriptions of *Plasmodium* spp. in deer since its first identification, recent research has rediscovered *Plasmodium* on ungulates in Africa, Asia, North America and South America, including Central Brazil. Here, a captive herd was evaluated in southern Brazil using light microscopy and PCR. DNA samples were tested for fragment amplification of two *Plasmodium* spp. genes: mitochondrial cytochrome b and small subunit ribosomal RNA.

**Results:**

All analyses were negative. However, the tests were performed on samples that were collected at a single time point, and parasitemia may fluctuate over the parasite’s life cycle. Thus, the possibility of occult infection cannot be ruled out. Despite the negative results of all of the methods applied, it cannot be categorically stated that these animals are free from *Plasmodium* sp. infection. Further monitoring and/or multiple sequential sampling may improve the success rate of detecting parasites. Moreover, although this survey of *Plasmodium* represents the first molecular study on ungulate malaria parasites from Southern Brazil, further analysis of samples from different ungulate species is important for characterizing the epidemiology of *Plasmodium* of these mammals in this region.

## Introduction

Malaria parasites have been described in a wide range of hosts in the Americas, including humans [1], monkeys [2], free-living birds [3], reptiles [4] and rodents [5]. Among non-primate mammals, *Plasmodium* species had been thought to be limited to the Old World. In particular, cervids had not been considered to be a vertebrate host due to the absence of parasites in blood smear investigations [6–8]. This idea was proved wrong in 1967 with the identification of a *Plasmodium* parasite (*P. odocoilei*) in a blood smear from a splenectomized white-tailed deer (*Odocoileus virginianus*) in Texas, United States of America (USA) [6, 9].

Surprisingly, recent reports have “rediscovered” malaria parasites in cervids and other ungulates throughout the world [10–14]. These studies have raised questions regarding the evolution of *Plasmodium* parasites, the cross-continental dispersion of these parasites and the role of ungulates as malaria reservoirs. Specifically, the recent identification of *Plasmodium*-positive deer in Central Brazil [14] led us to ask whether the presence of malaria reservoirs might extend to cervids from Southern Brazil.

A large herd of captive Brazilian dwarf brocket deer (*Mazama nana*) is protected in the Bela Vista Biological Sanctuary (BVBS), Foz do Iguaçu, Brazil. Red brocket deer (*M. americana*) and marsh deer (*Blastocerus dichotomous*) are also present. BVBS (25° 26′ 57″ S; 54° 33′ 18″ W) is a zoo and animal rehabilitation center located in a national protected area in southern Brazil and shares borders with Argentina and Paraguay. Cervids are kept in fenced areas that are covered and surrounded by native vegetation, making contact with free-living wild animals possible. Capybaras (*Hydrochoerus hydrochaeris*) are often seen moving freely through zoo areas. Our research group first reported *Plasmodium* sp. infection in capybaras that were captive animals at the BVBS, with findings of infection in 1/11 via microscopy and 3/11 via molecular testing [5].

In addition, the mosquito *Anopheles* sp., which is the vector implicated in *Plasmodium* spp. transmission, has been shown to have > 10% prevalence in this region, favored by the warm and humid climate [15]. Altogether, this region may provide conditions that are sufficient for a complete transmission cycle extending to wild mammals, particularly cervids.

In Brazil, the Brazilian dwarf brocket deer, red brocket deer and marsh deer have been screened for pathogens, with 27/31 (87%) infected by *Mycoplasma ovis* [16], 5/32 (15.6%) seropositive for *Toxoplasma gondii*, 2/32 (6.2%) positive for *Neospora caninum* and 1/32 (3.1%) positive for *Leptospira interrogans*. Additionally, all animals tested negative for *Brucella abortus*, bovine viral diarrhea virus, foot-and-mouth disease virus, infectious bovine rhinotracheitis, Eastern equine encephalitis, Western equine encephalitis and Venezuelan equine encephalitis [17]. This monitoring has provided important information for animal and public health and for conservation purposes, given that internationally, Brazilian dwarf brocket deer, marsh deer and red brocket deer have been considered vulnerable [18–20]. Thus, considering the recent reports about malaria parasites in cervids and other ungulates throughout the world, including Central Brazil [10–12, 14], we conducted a re-examination, using microscopic and molecular tools, of these 32 deer samples, searching for *Plasmodium* species.

## Main text

### Methods

#### Animals and blood collection

**Fig. 1:**
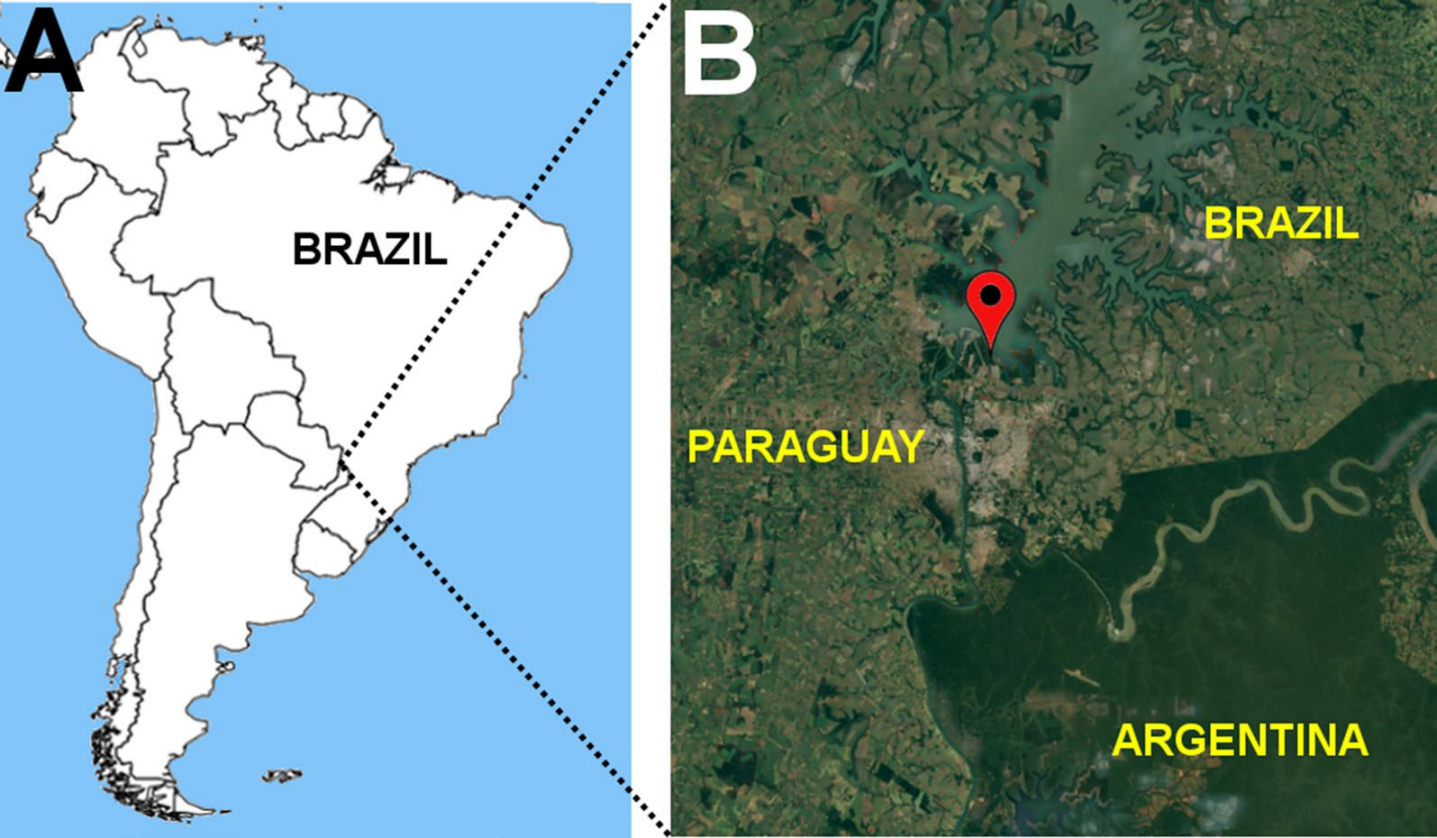
Origin of samples used in the study. **A** Location of Brazil in South America. **B** Location of Bela Vista Biological Sanctuary, Itaipu Binational Hydroelectric Power Plant, Foz do Iguaçu, Parana State Source: Modified from Google Earth

Blood samples from 32 cervids (22 *M. nana*, dwarf brocket deer; 4 *M. americana*, red brocket deer and 6 *B. dichotomus*, marsh deer) that were previously surveyed for other pathogens [16, 17] were included in this study. The animals were maintained in captivity at Bela Vista Biological Sanctuary, Itaipu Binational Dam, in Foz do Iguaçú, Paraná State, Brazil (Fig. 1). Since all animals were clinically healthy at the time of constraining and sampling, no preventive or curative treatment had been given to any of the captive deer surveyed herein. EDTA blood samples were collected by jugular venipuncture from the deer and were stored at − 20 °C.

Bela Vista Biological Sanctuary currently holds the biggest captive collection of Brazilian native deer worldwide. Death rates of Brazilian native deer due to constraint and sedation have varied from 10 to 100% [21]. Although no animal was lost as a result of the present survey, no further sampling has been performed.

This study was approved by Bela Vista Biological Sanctuary and the federal regulatory agency IBAMA (Brazilian Institute for the Environment and Renewable Resources) under the Protocol Number 22.158-2 (November 12, 2009) and was conducted in accordance with IBAMA’s ethical rules.

#### Microscopic examination

Peripheral blood smears were stained with May-Grünwald-Giemsa stain and were examined using optical microscopy at ×1000 (BX51, Olympus, Tokyo, Japan) [22].

#### DNA extraction and molecular analyses

DNA was extracted using a commercial kit (DNeasy Blood & Tissue Kit, Qiagen, Valencia, California, USA), in accordance with the manufacturer’s instructions. DNA quality was assessed through amplification of the mitochondrial cytochrome b (*cytb*) gene [23], using the primers L14841 (5′-AAA AAG CTT CCA TCC AAC ATC TCA GCA TGA TGA AA-3′) and H15149 (5′-AAA CTG CAG CCC CTC AGA ATG ATA TTT GTC CTC A-3′). PCR for *Plasmodium* spp. was performed using two previously described protocols: one targeting the small subunit RNA gene (SSU rRNA) [5, 24] and the other targeting the mitochondrial cytochrome b gene (*cytb*) of the parasite [25]. Both PCR protocols use universal primers for *Plasmodium* amplification, and the species of *Plasmodium* is determined by the similarity to the genomic sequences deposited in GenBank. The primers DW2 and DW4, used in *Plasmodium cytb* gene amplification [25], have also been used in other studies for *Plasmodium* screening from ungulates [10–12, 14]. All reactions were performed using positive and negative controls, and no contamination was detected.

## Results and discussion

**Fig. 2:**
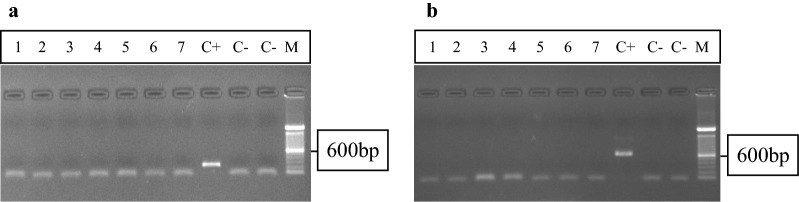
Agarose gel electrophoresis showing the size of amplified products by PCR with generic primers targeting a highly conserved region of 18S rRNA gene (**a**) or mitochondrial cytochrome b gene (**b**) of genus *Plasmodium*. (M) represents the molecular weight marker (100 bp DNA Ladder Invitrogen) (C−) negative controls (water) and (C+) a *Plasmodium* positive control (genomic DNA from *Plasmodium brasilianum*, Peru III strain). The numbers (1–7) show the results obtained for seven of all negative tested samples. **a** shows amplification product of ~ 240 bp only in the positive control as well as **b** shows amplification product of ~ 600 bp only in the positive control

No *Plasmodium*-like parasites were observed through direct examination of deer blood smears, and no DNA amplification was detected by means of PCR for either the SSU rRNA or the *cytb* gene (Fig. 2). However, negative findings obtained in this study may depend on the low parasitemia at the time of blood collection. Indeed, low parasitemia has been previously described in infected ungulates [6, 11, 12], and the incidence of infection has been as low as 0.003% [12] in molecular tests, with no detection of parasites in blood smears. Moreover, the life cycle of *Plasmodium* in vertebrate hosts may include a long-lived dormant stage in the liver, with sequestration of the parasite from the general circulation, causing very low parasitemia in the absence of an immunosuppressed state, as previously reported in water buffalos (*Bubalus bubalis*) [26]. Therefore, further studies are needed to confirm that examined deer are free from *Plasmodium* infection, despite the negative results obtained in the present study.

Nested PCR-based screening of white-tailed deer in 45 counties in the USA (all in the eastern region of the country) found that 41/308 (13.3%) of the animals were infected by *Plasmodium* sp. Conversely, previous studies have failed to detect *Plasmodium* in other ungulate species, including elk (*Cervus canadensis*), pronghorn (*Antilocapra americana*) and mule deer (*Odocoileus hemionus*) [11]. Additionally, previous PCR-based screening studies on other species of the order Artiodactyla, including sitatunga (*Tragelaphus spekii*), red river hog (*Potamochoerus porcus*) and water chevrotain (*Hyemoschus aquaticus*), also did not detect *Plasmodium* sp. [10]. More recently, the presence of ungulate malaria parasites in South America was reported for the first time in pampas deer (*Ozotoceros bezoarticus*) samples from Central Brazil [14]. However, in this same survey, samples from brown brocket deer (*Mazama gouazoubira*) and marsh deer (*B. dichotomus*) were negative for *Plasmodium* species [14], indicating that different deer species may present different levels of susceptibility to malaria.

Recent molecular clock estimates of *Plasmodium* spp. divergence have shown that the clade including *P. odocoilei* is likely to have diverged from other clades between 2.3 and 6 million years ago [11], thus suggesting that *Plasmodium* is an ancient parasite of deer. Hence, co-evolution of both species could have made deer a well-adapted host for the parasite. Other previous investigations on *Plasmodium* spp. in deer failed to detect the parasite [6–8], and some *Plasmodium* species have been discovered only after a host has been splenectomized [9].

The global health and ecological impact of malaria in wild animals is still unknown. Therefore, active surveillance providing epidemiological information regarding health status, mortality rates and geographic distribution of malaria infection in domestic and wild ungulates, both free-living and captive animals, including deer, is important to develop strategies for the management and control of malaria infection, thus improving the health and wellbeing of these animals.

## Limitations

The tests in this study were performed on samples that were collected at a single time point. Low parasitemia has been previously described for infected ungulates, and parasitemia may fluctuate over the parasite’s life cycle. Thus, the potential parasite life cycle at the time of blood collection may have had a direct impact on the negative findings. As the possibility of occult infection cannot be ruled out, despite the negative results of all of the methods applied, it cannot be categorically stated that these animals are free from *Plasmodium* sp. infection. Further monitoring and/or multiple sequential sampling may improve the success rate of detecting parasites. Moreover, although this survey of *Plasmodium* represents the first molecular study on ungulate malaria parasites from Southern Brazil, further analysis of samples from different ungulate species is important for characterizing the epidemiology of *Plasmodium* of these mammals in this region.
